# An Evolutionary Psychological Approach Toward BDSM Interest and Behavior

**DOI:** 10.1007/s10508-024-02881-x

**Published:** 2024-05-20

**Authors:** Michelle A. Larva, Markus J. Rantala

**Affiliations:** 1 INVEST Research Flagship Centre (Psychology), University of Turku, Assistentinkatu 7, N20014 Turku, Finland; 2 Department of Biology, University of Turku, Turku, Finland

**Keywords:** Biopsychosocial, Evolutionary psychology, BDSM, Sex differences, DSM-5, ICD-11

## Abstract

Bondage/discipline, Dominance/submission, and Sadism/Masochism (BDSM) have gained increased attention and discussion in recent years. This prevalence is accompanied by a shift in perceptions of BDSM, including the declassification of sadomasochism as a paraphilic disorder. Evolutionary psychology offers a unique perspective of why some individuals are interested in BDSM and why some prefer certain elements of BDSM over others (e.g., dominance versus submission). In this paper, we examine BDSM from an evolutionary standpoint, examining biopsychosocial factors that underlie the BDSM interests and practice. We articulate this perspective via an exploration of: proximate processes, such as the role of childhood experiences, sexual conditioning, and physiological factors; as well as ultimate explanations for power play and pain play dimensions of BDSM, highlighting the potential adaptive advantages of each. While BDSM may not be adaptive in itself, we examine the literature of sex differences in BDSM role preferences and argue that these preferences may stem from the extreme forms of behaviors which enhance reproductive success. In the realm of pain play, we explore the intersection of pain and pleasure from both physiological and psychological perspectives, highlighting the crucial role of psychological and play partner factors in modulating the experience of pain. Finally, we encourage future research in social sciences to utilize evolutionary frameworks to further explore the subject and help alleviate the mystification surrounding BDSM. This multifaceted exploration of BDSM provides valuable insights for clinicians, kink-identified individuals, and scholars seeking to understand the evolutionary perspectives of human sexual behavior and preferences.

## Introduction

The abbreviation BDSM, previously referred to as sadomasochism (SM), encompasses Bondage and discipline, Dominance and submission, and Sadism and Masochism. This term usually refers to physical, psychological, and sexual role-play involving power exchange between consensual participants (De Neef et al., 2019). Defining BDSM, or “kink,” practice or play more precisely is challenging due to its heterogeneity in practices, but often it involves power play, physical restraint, strong sensory experiences, as well as enjoyment of physical and psychological control and strong stimuli (Paarnio et al., 2023). There are significant individual differences in motivations for BDSM practice, which practices are regarded as BDSM, and how pleasurable individuals find these practices to be; sexual behavior that one individual would regard as mainstream or “vanilla” may be kinky for another. BDSM practitioners can be divided into groups based on preferred roles, the most common of which are those who want to be in control (also referred to as the dominant partner), those who want to give up control (the submissive partner), and those who prefer both roles (i.e., “switch”) (De Neef et al., 2019; Paarnio et al., 2023).

Many misconceptions surround BDSM. The first of these to address is its equation with violence. As a form of consensual play between adults, most often involving forethought and prior agreements, BDSM is distinct from violence. The consensual aspect often includes the identification of a safe-word, which is intended to immediately stop the play when spoken, and the disclosure of boundaries. Another common misconception related to BDSM regards pain, or strong stimuli, being at the center of BDSM. Yet, pain plays a much less central role in BDSM interactions and is more so used as a tool to achieve power dynamics in an erotic context (Cross & Matheson, 2006; Gebhard, 1969). Finally, while BDSM is most often associated with sex, explicitly sexual behaviors involving genitalia are not necessarily included in all BDSM practice (Ambler et al., 2017).

Elements of BDSM and its practice are not uncommon. The prevalence rates for BDSM practice and interest are highly varied depending on the definitions used (i.e., what constitutes as BDSM) and the sample surveyed (Paarnio et al., 2023). For example, one conservative estimate came from an Australian sample of the general population, where 2% of men and 1.4% of women reported having engaged in BDSM in the last 12 months (Richters et al., 2003). A more liberal and recent estimate from a sample of the general Belgian population revealed that 47% of respondents had ever performed at least one BDSM-related activity and another 22% reported having BDSM-related fantasies (Holvoet et al., 2017). What is relatively consistent across the literature, however, is that prevalence rates in BDSM activities tend to be higher for young and non-heterosexual adults, and that BDSM interest and fantasies are more prevalent than the practice itself (De Neef et al., 2019; Paarnio et al., 2023).

It is difficult to empirically capture the full scope of BDSM practices. One such attempt by Alison et al. focused on a sample of SM-practitioners in Finland (Alison et al., 2001). They identified BDSM-related behaviors belonging to four clusters of sexual scripts, each with its own psychological significance. The four clusters consisted of: hypermasculinity (e.g., dildo use, anilingus); administration and receiving of strong stimuli; physical restriction; and humiliation. Due to the limitations of the sample, these groupings are likely not representative of the broader BDSM community; nevertheless, they offer a representation of the heterogeneity of interest and practice in the BDSM community. Correspondingly, participants in this study preferred a certain type of SM-behavior, and few were interested in all activities. For example, heterosexual males practiced more humiliation of the partner than homosexual males, whereas homosexual males were more likely to practice SM-behaviors grouped into the hypermasculinity category (Alison et al., 2001).

Another useful representation of heterogeneity in BDSM comes from a more recent study conducted by Weierstall and Giebel (2017). With the assistance of members from the German SM community, they developed the Sadomasochism Checklist—an assessment tool for measuring an individual’s engagement in SM practices. The 24-item checklist consists of a submission scale and a domination scale assessing six different types of practices: soft play, domination/submission, beating, sex toys, breath play, and play with bodily fluids (De Neef et al., 2019).

Particularly in the recent decade, BDSM has seen increased exposure across mainstream media, growth in scientific literature, and thus an increased awareness of the topic in popular culture overall. Despite this progression, BDSM often continues to be misunderstood. Its historical and continued pathologization subjects the BDSM community to misconceptions and stigmatization (De Neef et al., 2019). The DSM-5 classifies sadomasochism as a mental health disorder if it results in daily stress for or issues in everyday functioning of the individual (American Psychiatric Association, 2013). However, according to the current evidence available, stress or other problems are rarely associated with SM (see Moser, 2009; Ten Brink et al., 2021; Wismeijer & van Assen, 2013). For example, in a study conducted in the Netherlands with 902 participants who practiced SM and 432 participants who did not, Wismeijer and van Assen found that SM was not associated with psychological issues. In fact, SM-practitioners were less neurotic, more open, less sensitive to rejection, and more securely attached to their loved ones. Those who practiced SM also scored higher on self-reported emotional and physical well-being compared to those do did not practice SM. Notably, due to the correlational nature of the data, causal conclusions cannot be drawn based on these studies.

Sociocultural influences and trends are undeniably salient contributors to human behavior and interests, as in the case of BDSM. A recent study by Schuerwegen et al. (2023) investigated cultural differences in BDSM interests, fantasies, roles, and practices, and found cultural variation particularly in age of onset of BDSM fantasies (earlier among Europeans) and the public nature of BDSM practitioners’ play (more frequent among North Americans). Further research into this area is warranted, particularly examining non-WEIRD populations. Additionally, exposure to BDSM portrayals in popular culture, such as movies like *Fifty Shades of Gray* (Taylor-Johnson, 2015), erotic novels (Kimberly et al., 2018), or trending TikTok videos can create proximate pathways into BDSM interest and practice (Walker & Kuperberg, 2022). These sociocultural contributors coexist with biological influences, which can include adaptations shaped by natural and sexual selection over the course of millennia. We can examine such influences on human cognition and behavior more closely through the framework of evolutionary psychology. Applying an evolutionary psychological framework to the literature on BDSM can expand our understanding about the underlying factors and evolutionary context surrounding BDSM-related behaviors, as well as how these aspects may surface and develop across the individual’s lifespan. The focus of this paper concerns some of the proximate (i.e., how the behavior develops psychobiologically and the mechanisms behind it) as well as ultimate explanations (i.e., regarding its evolutionary history and adaptive significance; Nesse, 2019) for BDSM.

In mind and body, Homo sapiens have been shaped by the same, inevitable force of nature which shapes all species—natural selection. Natural selection selects against genes or alleles within a population which reduce reproductive success (i.e., one’s ability to pass on genes to the next generation), favoring genes which enhance reproductive success. At first glance, then, it may be perplexing to consider genes that code for certain preferences or inclinations that could be associated with BDSM. What would such inclinations have to do with reproductive success? While there are certainly differences between having BDSM-related preferences, engaging in BDSM-related acts, and being a BDSM practitioner, it is apparent that a notable portion of the population of the human species engages in these behaviors. It is therefore reasonable to consider that BDSM interests and behaviors may have proximate and ultimate explanations. To thoroughly assess BDSM preferences and behavior, we address the topic at proximate and ultimate levels of analysis: proximate physiological mechanisms, ontogeny, evolution, and ultimate reproductive value. These “levels of analysis” proposed by Tinbergen (1963) are used to understand and describe some feature of an organism: How does it work? (physiology/mechanisms). How does it develop over the course of an individual’s lifespan? (ontogeny). How did the feature evolve over the history of a species? (evolution or phylogeny). What is it for? (ultimate reproductive value or function) (Luoto et al., 2019; Nesse, 2019).

## Proximate Explanations

### Physiology

Few studies have examined the underlying biological processes behind BDSM interactions, and further research is needed to comprehensively understand the driving mechanisms and physiology underlying the diverse landscape of BDSM (De Neef et al., 2019; Wuyts et al., 2020). For example, to the best of our knowledge, researchers have yet to observe BDSM interest or practice across the menstrual cycle, examining whether female hormonal changes may be associated with either. Considering previous studies have observed fluctuations in sensitivity to pain and preferences for certain behavioral displays at different time points in the menstrual cycle (e.g., see Riley et al., 1999, and Gangestad et al., 2004, respectively), one opportunity for future research into the physiology of kink could be the exploration of hormonally mediated variations in BDSM-related interests, fantasies, and practices.

De Neef et al. (2019) as well as Wuyts and Morrens (2022) have systematically reviewed studies that have uncovered biological processes behind BDSM. Both reviews identified and discussed evidence related to hormonal and neurological workings involved in BDSM practice, mainly during and post-BDSM play. These reviews offer valuable insight into these processes. De Neef et al. (2019) also presented a novel, preliminary, biopsychosocial model for BDSM interests—displaying the biological, psychological, and social factors contributing to a multi-dimensional spectrum of BDSM interests. We want to build on these works by addressing two additional, potential, physiological mechanisms involved in BDSM interests and behaviors.

Firstly, it should be highlighted that pain and sexual pleasure share common pathways and systems between the body and the brain. For one, when we experience pain, our brains activate their natural opioid system; this is the body’s evolved attempt to block the sense of pain, but in can also have a euphoric effect. Further, neurological research has identified that the brain’s reception of signals for both orgasm and pain run through the spinothalamic system. In one study, neurologists Berić and Light (1993) observed that—in patients with spinal cord injuries in which the spinothalamic tract was severed, although the patients had not lost all sensation in these areas, they lost both the sense of pain and the ability to reach orgasm. In similar cases documented by Elliot (1969, as cited in Komisaruk & Rodriguez del Cerro, 2021) where the spinothalamic pathway was severed surgically, when patients regained pain sensation months later, they also regained the ability to orgasm. It has also been previously pointed out that the body’s physiological response, particularly within the autonomic nervous system, is similar for nociceptive pain and sexual arousal (e.g., pupil dilation, elevated heart rate, skin conductance, respiration; Sutter, 1954, as cited in Wuyts et al., 2020).

Wuyts et al. (2020) experimentally examined several biomarkers within BDSM interactions in a small pilot study of Flemish BDSM and non-BDSM practitioners. They experimentally and biologically confirmed what numerous psychometric and qualitative studies have previously asserted: pain from BDSM interactions elicits a physiological pleasure response in submissives. This study did not, however, find as strong of changes in dominants’ pleasure biomarkers. Based on post hoc analyses, Wuyts et al. suggested that this may be due to power dynamics playing a larger role in the moderation of dominants’ pleasure in BDSM interactions.

Secondly, when considering explanations at the proximate level, manipulators or pathogens must also be taken into consideration. Countless animal studies have reported mechanisms by which a manipulator will alter its host in ways that yield fitness gains for the manipulator itself. Many (such as Berec & Maxin, 2014; Borráz-León et al., 2022; Sarafin, 2020) have raised the need for further empirical investigation into the potential manipulative mechanisms of sexually transmitted organisms (STOs) in humans. While rarely studied, the concern is that STOs may manipulate the behavior and/or appearance of its host in order to boost the host’s fitness, which produces fitness gains for the STO. In such cases, it is worth asking whether STOs could impact sexual behavior in ways that translate to attraction toward BDSM in affected humans.

Flegr and Kuba (2016) investigated the associations between various sexual interests, sexual behaviors, and toxoplasmosis, a common infection caused by the protozoan parasite *Toxoplasma gondii*, which is understood to alter the behavior of its host’s sexual arousal pathways and defensive behavior to increase the brain parasite’s own fitness (Borráz-León et al., 2022; House et al., 2011). Although Flegr and Kuba’s study comes with considerable limitations related to data collection, it offers us a glimpse into one of many potential mechanisms and manipulators at play in human sexual preferences. Participants with latent toxoplasmosis indicated greater sexual attraction/arousal to bondage sex and feelings of fear, danger, pain, powerlessness, or humiliation (generally, both their own and partner’s) than noninfected participants (Flegr & Kuba, 2016). Interestingly, these attractions did not translate to consumption of BDSM-porn nor to BDSM practice. Indeed, *Toxoplasma*-infected participants reported engaging in BDSM activities less often compared to uninfected participants. The researchers suggested that this incongruence between attraction and practice could be a result of the disruptive effects that *Toxoplasma* can have on dopaminergic pathways in the host’s brain, which could reduce the individual’s novelty-seeking attributes and thus, translate to less engagement in BDSM activities. Toxoplasmosis is also associated with higher testosterone levels in males (Borráz-León et al., 2021), and while testosterone is implicated in sexual motivations and social dominance, its link to dominance in the BDSM context remains unconfirmed (Wuyts & Morrens, 2022).

### Ontogeny

Next, we address the question: what might have occurred over the course of an individual’s development that contributed to BDSM interests in the first place? In the following sections, we will address two interconnected, developmental processes that may play a role in some individuals’ BDSM-related sexual interests, namely sexual conditioning and sexual imprinting. Some say sexual conditioning and sexual imprinting share the same mechanism. However, we argue that these mechanisms should be considered separately given that they serve distinct adaptive functions. A sexual stimulus is not a requisite factor in sexual imprinting and would take place during early childhood; in contrast, sexual conditioning hinges on the presence of a sexual component, whereby pleasure becomes associated with a particular stimulus.

#### Sexual Conditioning

In sexual classical conditioning, one learns to associate a nonsexual stimulus with sexual pleasure. A range of atypical sexual behaviors and interests can come about in this way, and likewise, some forms of BDSM may overlap with sexually conditioned kinks or fetishes (Breslow et al., 1985). Empirical observations of sexual conditioning and imprinting have, for well-understood ethical reasons, been primarily conducted in animal studies. Popular examples of sexual conditioning studies have been conducted with rats; for example, researchers observed that if a young and sexually inexperienced male rat was given the opportunity to mate with females that smell like almond or lemon, an olfactory nonsexual stimulus, these males preferred to mate with females with these scents also later in life (Kippin et al., 1998). Analogously, male rats that did not undergo this conditioning preferred to copulate with females that were unscented. In a similar study, simply smelling the sexually conditioned scent was observed to raise sex hormone levels (Graham & Desjardins, 1980). Conditioned males even exhibited less sexual interest in unscented females, more frequently attempting to copulate with scented females even when they were not in estrus (Kippin & Pfaus, 2001). Similar findings have been observed in studies conducted with our fellow members of the primate order, including one in which young male marmosets, sexually conditioned with lemon-scented female marmosets, experienced erections from the mere smell of lemon one week following the conditioning (Snowdon et al., 2011). Sexual conditioning has also been demonstrated in rat experiments where nonsexual stimuli did not require intercourse to produce a “kink” for those stimuli, but in which synthetic clitoral stimulation and even oxytocin injections achieved the same result (Pfaus et al., 2012).

Sexual conditioning can also occur with environmental and somatosensory cues; for example, this was observed in a study in which male rats being placed into a chamber where they previously copulated elicited an erection, whereas being placed in another chamber did not (Sachs & Garinello, 1978). Arousal in response to certain clothing, used in conjunction with early sexual experiences, can also be sexually conditioned. This was well-demonstrated in another experiment conducted with rats in which researchers (Pfaus et al., 2013) divided sexually naïve male rats into two groups; males in one group wore a jacket during their first copulatory experience with a receptive female, and males in the other group had their first copulatory experience with a receptive female without wearing a jacket. When the jacket-trained males were given the opportunity to mate with females without their jackets, they had three times as many issues with ejaculation and took eight times as long to reach intromission as when mating while wearing jackets. Stated otherwise, the male rats developed something of a fetish for jackets.

To better understand how sexual conditioning can occur in our own species, we can also consider a handful of human studies conducted in the 1960s and 1970s (for examples, see Kimura et al., 1990; Marquis, 1970; Quinn et al., 1970; Rachman, 1966; Rachman & Hodgson, 1968). In one of such studies by Rachman and Hodgson, five male participants were conditioned by being shown nude photographs of women wearing knee-length boots. The researchers tested whether this conditioned stimulus (the boots) would not only elicit arousal but also become generalized to women’s shoes in general. Following the conditioning, three out of the five were indeed aroused by the sight of varying pairs of women’s shoes. While most of these studies lacked in robust sampling and meeting adequate experimental conditions, they offer a demonstration of how sexual kinks in humans could develop via conditioning. While unspecific to kink or BDSM, other methodologically-sound human sexual conditioning studies have also been conducted in the more recent decades and provide compelling evidence of, but also complications associated with, human sexual conditioning (see Hoffman, 2017).

Unconventional sexual preferences in humans can appear as early as adolescence (American Psychiatric Association, 2013). Therefore, it is possible for sexual conditioning to take place as early as childhood and youth, not enforced by intercourse but resulting from a nonsexual cue or stimulus paired with rewarding sexual pleasure (i.e., the individual learns to associate the conditioned stimulus with pleasurable sensations, resulting from some form of genital stimulation or masturbation). Sexual conditioning certainly cannot explain every BDSM-related preference, and one caveat to this suggested mechanism is that sexual conditioning should imply a sensitive period of development, which to our best knowledge has not been documented in humans. Still, sexual conditioning is one potential mechanism by which individuals may develop an interest in BDSM. This could apply to various aspects of BDSM preferences and practices if there is—most likely repeated—exposure to the BDSM-element paired with a reward. An example of this was given by one BDSM practitioner, who in early pubescence would view websites that sold chastity cages while masturbating. In adulthood, almost 20 years later, chastity cages continued to act as a model for their sexual encounters and BDSM practice (Walker & Kuperberg, 2022).

#### Sexual Imprinting

A well-known example of sexual imprinting comes from the Austrian zoologist, ethologist, and ornithologist, Lorenz (1903–1989), and his gooselings. Chicks and goslings had long been known to imprint on the first moving subject they see, typically their mother, and to follow this subject throughout their youth, thus increasing their chances of survival. Yet, Lorenz discovered that in the absence of the mother, graylag goslings could imprint on a researcher or an object, such as the boots he wore. He also found that this maternal imprinting was possible during a 13–16 h sensitive period, after which even the true mother no longer sufficed for the offspring. Interestingly, Lorenz noticed that the goslings which had imprinted on him also displayed sexual interest toward humans upon reaching adulthood.

Generally in nature, the caregiver belongs to the same species as its young. Therefore, an ultimate explanation for sexual imprinting is that it enables the animal to know what a potential mate should look like. In other words, this sort of learning ensures that the animal would mate with its own species and hence promotes reproductive success. The studies that followed Lorenz’s finding demonstrated that—in many species of birds, mammals, including primates—sexual imprinting can extend beyond the conspecific caregiver, shaping the individual’s mating preferences upon reaching sexual maturity, in which case the reproductive advantage is not realized. Zoo caretakers are particularly prone to sexual advances by the animals they care for (Lorenz, 1931, 1937; Nicholls, 2010). Over the years, zoologists have learned that preventing inconvenient sexual imprinting requires some effort from the caretakers; nowadays, for example, young pandas’ caretakers can be seen dressed as their conspecifics so as to prevent sexual imprinting on humans (Nicholls, 2010). In many species, sex differences exist in sexual imprinting sensitivity and stability; an example of this is presented by Kendrick et al. (1998), who observed that the effects of imprinting on foster species persisted several years in male sheep and goats, but not female sheep and goats.

Not all species are equally sensitive to sexual imprinting, some possessing strong innate mate preferences and unswayed by caregiver characteristics. As sexual imprinting is quite commonly observed throughout the animal kingdom, however, finding human young an exception would be a remarkable oddity. Indeed, it appears that sexual imprinting can occur in humans to some extent. The influence of childhood experiences and parental characteristics on our later behavior in adulthood is indeed at the core of various developmental psychological theories. Empirical observation of the effects of sexual imprinting on the development of human sexual preferences are unethical and thus impossible to carry out in the same way as with other species. Instead, the literature available on sexual imprinting in humans is based in correlational studies comparing childhood experiences of parental or sibling characteristics with partner preferences and/or choices later in life. For examples, see Jedlicka (1980, 1984) for ethnic groups; Little et al. (2003) and Wilson and Barrett (1987) for eye and hair color; Perrett et al. (2002) and Wilson and Barrett (1987) for age; Rantala et al. (2010) for hairiness. Unfortunately, most of these studies concern heterosexual attractions and none have accounted for bisexual, genderqueer, trans, or intersex identities for example—a notable limitation in the current literature. Overall, there is some evidence to support the notion that our mate preferences, sometimes even our chosen romantic partners, can reflect our parent of the sex we are attracted to, either in nature or appearance.

Several studies have investigated similarities in partner and parent appearance. Sexual imprinting would imply that individuals would be most attracted to mates with similar physical features to the parent (as experienced during childhood) of the sex they are attracted to. Valentova et al. (2017) found that heterosexual men’s and non-heterosexual women’s partner preferences for physique were positively correlated with the physique of their mothers. In another study of heterosexual women and homosexual men, father’s physique was observed to be mildly, positively correlated with preferred physique (Sterbova et al., 2018). The effect was strongest in straight women who had had a positive and warm relationship with their father. Still, this preference did not translate in practice to their actual romantic partner’s physique. However, some research examining facial features has observed sexual imprinting translating to actual partner choice, sometimes moderated by quality of the parent–child relationship (Kocsor et al., 2016; Vukovic et al., 2015; Wiszewska et al., 2007). For both sexes, findings are more consistent regarding emotional closeness as a predictor of opposite-sex parent resemblance to one’s mate preferences (Vukovic et al., 2015; Wiszewska et al., 2007).

One example of sexual imprinting in humans on non-physical traits comes from Gyuris et al. (2010), a study in which researchers examined correlations between heterosexual individuals’ spousal and parental personality. For women, the researchers did not identify significant, direct associations between spousal and parental personality traits. For men on the other hand, conscientiousness scores between men’s wives and both parents were positively correlated. Further, the researchers found a tendency toward correlation of the men’s mothers and spouses on conscientiousness in men who received little love from their mother. Sons who experienced high levels of rejection also had wives who scored significantly similarly to their mothers on emotional stability. However, this study also revealed some associations between spouses’ and same-sex parents’ personalities, particularly when the quality of the parent–child relationship was accounted for. These findings highlight the nuance of sexual imprinting in humans. Other studies have also examined associations between parental factors and some forms of paraphilia, including capnolagnia (smoking fetishization; Aronsson et al., 2011) and maiesiophilia (a fetishism concentrated on pregnant or breastfeeding women; Enquist et al., 2011). In the latter example, upon closer examination, researchers observed a higher prevalence of maiesiophilia in men whose mothers had been pregnant while they were 1.5–5-years-old; this finding provides us a hint of which age range may be a sensitive period for sexual imprinting in humans (Enquist et al., 2011).

It is plausible that sexual imprinting could partially explain some cases of kinky interest and practice. An individual might sexually imprint on their parent/caregiver (most likely of the sex[es] they are attracted to) along with their qualities, which would later translate to their sexual preferences and contribute to BDSM-related practice. In other words, the adaptive function of sexual imprinting may come with the side effect of developing an attraction for the qualities of the individuals they were raised by; and if these qualities are fitting for BDSM practice, the individual might find themselves also attracted to BDSM.

Further research is needed to better understand the potential relationship between BDSM interest/engagement and parental disposition, parenting style, and parental couple power dynamics (De Neef et al., 2019) experienced in childhood. For example, could it be that heterosexual males’ BDSM role identity as dominants would correlate more strongly with their mothers having a submissive disposition as opposed to a dominating disposition? Drawing from the available evidence, we would hypothesize that males are more sensitive to the influences of sexual imprinting. This would be supported by evidence from Breslow et al. (1985), who found that males’ age of first interest in BDSM was earlier (i.e., already during childhood) compared to that of females, and that males even reported these interests as “natural from childhood” (p. 310) more often than female participants. We also affirm the proposition made by Gyuris et al. (2010) that parental investment mediates the potency of sexual imprinting—in this case, whether parental qualities and individuals’ BDSM preferences are related.

Although imprinting is a distinct process from attachment, research on the potential role of attachment style (a by-product of early life parent–child interactions) in BDSM preferences may offer clues as to how a parent–child relationship can impress upon one’s sexual interests in later in life. Santtila et al. (2001) have offered evidence that the mother-son relationship significantly correlates with BDSM orientation. In a Finnish sample of SM-identified men, men who exclusively practiced sadism were more likely to exhibit avoidant attachment with their mothers and less likely to exhibit secure attachment with them; the reverse association was found for exclusive masochists, who were more likely to exhibit secure attachment and less likely to exhibit avoidant attachment with their mothers (Santtila et al., 2001). However, Wismeijer and van Assen (2013) did not report significant differences between the attachment styles of dominant, submissive, switch, and non-BDSM identifying individuals, although their results trended toward favorable attachment scores for dominants. More recently, Ten Brink et al. (2021) found that participants with a BDSM identity were more securely attached than those who were uninterested BDSM.

Many have been curious about whether BDSM interest in adulthood is related to childhood trauma or abuse, proposedly as a result of sexual imprinting. Yost and Hunter (2012) collected qualitative data from kink-identified individuals and found that a few identified past childhood abuse as part of their narratives of kink sexuality. At least three other, quantitative studies have found correlations between past childhood abuse and engagement in adult kink/BDSM practices (Abrams et al., 2021; Hopkins et al., 2016; Nordling et al., 2000). Contrary to these findings, however, a nationally representative survey in Australia did not find a statistically significant correlation between sexual abuse or coercion experienced before age 16 and BDSM participation (Richters et al., 2008). Notably, this study by Richters et al. did not inquire specifically about early childhood sexual trauma (i.e., during the sensitive period proposed above). Similarly, Ten Brink et al. (2021) did not find an association between childhood trauma and BDSM interest when inquiring about physical abuse experienced during childhood in general (age unspecified). Based on these findings, definitive conclusions are unwise to draw. Further research is needed to examine the possible sexual imprinting interactions between BDSM interest and engagement and exposure to or experience of abuse at varying points during childhood.

Based on studies accounting possible sexual imprinting in humans, it is difficult to state for certain whether these findings truly reflect a developed preference, versus how readily we might forgive the same, perhaps even distressing characteristics of our parents. It is also difficult to eliminate the possibility that partner and opposite-sex parent similarity in appearance would be the result of genetic inheritability (Rantala & Marcinkowska, 2011). However, one study of Hungarian women who were adopted as children yielded results that were also consistent with sexual imprinting on their adoptive fathers, moderated by the quality of the father-daughter relationship (Bereczkei et al., 2004). Additionally, given some of the mixed findings of sexual imprinting in humans, it is worth considering that this mechanism may occur more on the basis of parental investment rather than sex alone (Gyuris et al., 2010). Further research is needed to clarify how these processes play out. Nevertheless, much of the evidence points toward humans’ capacity to sexually imprint on various attributes of their parents/caregivers, which could translate to BDSM-related interests as well.

## Ultimate Explanations

In the following sections, we offer ultimate explanations for two, non-mutually exclusive dimensions of BDSM behaviors, power play and pain play (De Neef et al., 2019). Within this section, we do not engage in an in-depth exploration of BDSM from a phylogenetic perspective. Although a comprehensive evolutionary approach should encompass this dimension, the lack of empirical data hinders us in this regard. We acknowledge the potential for such an endeavor; however, given the predominance of BDSM research conducted within WEIRD cultural contexts, we are currently constrained in our ability to provide an evidence-based analysis at this level. Certainly, dominance- and submission-like behavior can be found in non-human species; for example, male viverrids (e.g., civets) and mustelids (e.g., weasels) are known to bite females during copulation (Le Boeuf & Mesnick, 1991). Yet, as with the previous example, there is a plethora of nuance to identifying BDSM-related behaviors in primates and other mammals—a complex subject that warrants a dedicated manuscript of its own.

Therefore, our emphasis is on investigating potential adaptive advantages. For example, why might some individuals find submission arousing while others are more aroused by dominance? What role does an individual’s sex or gender identity play in BDSM preferences? Are some aspects of BDSM associated with a potential reproductive advantage?

### Power Play

Significant sex differences can be observed in role preferences within BDSM (i.e., whether one prefers the role of the dominant or the submissive, or both). According to a study conducted in the Netherlands, about every three in four (75.6%) women who practiced SM reported that they preferred to be in the role of the submissive, and only 8% preferred to be the dominant (Wismeijer & van Assen, 2013); 16.4% of women identified as a switch, or alternating between the dominant and the submissive. Of the SM-practicing men in this sample, only one in three (33.4%) indicated a preference to occupy the submissive role, whereas playing the role of the dominant was preferred by 48.3% of the men in the sample. Although many men as well as women enjoy both roles, the research indicates that, compared to men, women experience more pleasure on average from playing the role of the submissive (Joyal & Carpentier, 2017). Men on the other hand tend to be more aroused by playing the role of the dominant (Jozifkova, 2013).

It is possible that this sex difference in BDSM preferences would result from sex differences in preference for masculine and/or feminine traits and behaviors. For the purposes of this paper, we treat objectively determined masculine or feminine behavior as such that is assessed according to average differences in the behaviors of males and females within a given culture. Others before us, such as Jozifkova (2013) and Brown et al. (2020), have drawn the connection between such reproductive strategies and dominance and submission. Females throughout the animal kingdom are known to prefer dominant males (i.e., broadly defined as males displaying behaviors that signal the ability to influence others either through social or physical means [Keating & Bai, 1986, as cited in Batres et al., 2015]) as sexual partners. This can be attributed to the idea that male dominance is a signal of good genes and health (Rantala & Kortet, 2004). In humans, masculinity of the male face and body (i.e., referring to sexual dimorphism) has been found to correlate quite robustly with stronger immune functioning (Luoto et al., 2019; Rantala et al., 2013). There is also some evidence that masculinity in human males is also related to general health (Rhodes et al., 2007). Additionally, in a study where researchers experimentally examined facial sexual dimorphism and sexual preferences, men whose faces appear more masculine were perceived to behave more dominantly than less masculine-appearing men (Batres et al., 2015).

Spanking, pain administration, or playing the role of a sex slave master for example, can be seen as assertions of dominance over another individual and as hypermasculine behavior (Weinberg et al., 1984). Extensive evidence spanning various mammalian species suggests that variations in prenatal exposure to sex hormones lead to divergences in brain structure, particularly in the hypothalamic preoptic area (SDN-POA in non-human animals, INAH3 in humans), potentially influencing predispositions toward masculinized or feminized sexual behavior and preferences (see Luoto & Rantala, 2022). Studies in cats, ferrets, rats, and guinea pigs have shown that surgical lesions to SDN-POA not only causes males to prefer same-sex individuals as mates, but also feminizes their sexual behavior (Cherry & Baum, 1990; Hart & Leedy, 1983; Hennessey et al., 1986; Kondo et al., 1990; Olster, 1993; Paredes & Baum, 1995; Rodriguez-Sierra & Terasawa, 1979). For example, males reduce their mounting behavior and begin to present lordosis (Hennessey et al., 1986; Olster, 1993). In humans, this could suggest that the analog area, INAH3, may not only play a role in one’s sexual orientation but also one’s favored role in coitus (i.e., being a “top” or “bottom” in the case of homosexuality). As such, it may also be associated with preferences for submissive versus dominant roles in BDSM. It may be that, accounting for plenty of within-group variation by sex, in utero exposure to estrogens and/or androgens may predispose individuals to finding certain BDSM behavior on a hypermasculinized/hyperfeminized spectrum of preferences attractive. Sexual masculinization of this brain region could predispose an individual to sexually prefer engaging in dominant behavior, whereas sexual feminization may be associated with a preference for submission in BDSM play.

Correspondingly, we predict that an individual with masculinized morphology in the hypothalamic preoptic area of the brain would find being in the dominating role more sexually arousing than what is presented sex-typically. This prediction is supported by studies that suggest heterosexual men often prefer the dominant role over the submissive role (Nordling et al., 2006) compared to homosexual men, who have been shown to display similar sex-differentiation (i.e., feminization) in cerebral morphology (Savic & Lindstöm, 2008; Nordling et al., 2006). In the sample of SM-practitioners in Finland mentioned earlier (Alison et al., 2001), heterosexual males practiced more humiliation of the partner than homosexual males, whereas homosexual males were more likely to practice SM-behaviors grouped into the hypermasculinity category. These included behaviors such as cock binding, use of dildos, watersports (urinating or being urinated on), rimming (analingus), enemas, and catheterization. During SM play, gay males were also more likely to administer stronger stimuli and in more concrete ways compared to heterosexual SM play, where pain administration was more symbolic (Alison et al., 2001). Homosexual men have been shown to prefer masculine features in their sexual partners (Glassenberg et al., 2010; Shiramizu et al., 2020); thus, gay males may find their sexual partners’ hypermasculine behavior (i.e., domination) arousing. At the time of this publication, research has yet to empirically examine the relationship between masculinized/feminized structures of the brain and one’s preference for being the dominant or the submissive. Further research is needed in order to confidently declare this association and determine whether the stereotype of the masculine-behaving dominatrix (or the femdom, a woman who takes the dominant role during BDSM play) can hold true. Along this line of thinking, for example, we would predict that kink-identifying, gynephilic, butch-identifying females are more likely to identify as dommes compared to those who are femme-identifying (Luoto et al., 2019).

The existence of switch BDSM identities does not necessarily mean that BDSM role preferences cannot be the result of the sex-typicality of one’s brain. Indeed, a portion of individuals fall somewhere in the middle of the gender spectrum, or rather outside of the gender binary, which would offer a natural explanation for a lack of preference for a particular role. We propose that switch-identified individuals may be more likely to also identify as non-binary. To the best of our knowledge, neuroimaging studies have yet to examine individuals with non-binary gender identities. The associations of neurological sex-typicality, gender identity, sex, and one’s sexual preferences, including BDSM identities, need further examination. Finally, this evolutionary psychological explanation also does not imply that human developmental experiences, societal, or cultural factors have no influence the practice of BDSM; it merely helps to explain the likelihood of role preferences.

### Pain Play

Over the course of our evolution, we have developed the ability to feel pain for the adaptive purpose of avoiding injury and things that cause pain (Williams, 2016). A good example of the importance of the pain sense to our survival and well-being is the case of Congenital Insensitivity to Pain and Anhidrosis (CIPA). CIPA is a rare disorder in which an individual is physiologically unable to feel pain, and many who suffer from it die by the age of 30 from injuries and unnoticed illnesses (Sztriha et al., 2001). On the other hand, we have also developed a pleasure system, which rewards us by producing sensations of pleasure from things that improved our ancestors’ odds of passing on their genes, such as sex and eating, since pleasure motivates us to repeat behaviors. Thus, animals are generally motivated to avoid pain and to seek pleasure, but this paints the traditional picture of pleasure and pain as contrasting forces. Today, we know this not to be the case, as findings from both pain and reward research domains suggest significant overlaps in the neural structures responsible for processing both distressing and enjoyable sensations (Leknes & Tracey, 2008). Thus, to address pain play from an ultimate perspective, a more nuanced understanding is necessary.

Many SM-practitioners assert that power play, addressed in the previous section, occupies a more central role in BDSM than administration or experience of pain during pain play (e.g., the administration of pain can serve to elevate the dominant’s status of power). But in cases where pain is still somewhat central to the play, it is important to consider the multifaceted nature of these experiences within BDSM. To enhance our understanding, it is crucial to acknowledge the distinctions between reward and pleasure. Namely, there is an intricate interplay in human hedonic experiences between reward and pleasure, generated in distinct brain systems (Berridge & Kringelbach, 2015). Understanding these nuances can shed light on the motivations and satisfactions of individuals engaged in BDSM practices. For example, some practitioners of SM report that pain itself is not arousing or rewarding for them, but rather the idea of pain and the threat of pain can be a main source of pleasure (Moser & Kleinplatz, 2007).

Many individuals who practice SM have reported that the stress relief they experience from SM play is one major motivation behind their practice (Pitagora, 2017). Stress is met with a fight or flight response (Rantala et al., 2018), and an individual who is bound or dominated is not able to fight, and must submit to the will of another. Thus, one can imagine that in this situation, the stress system shuts down, just as in individuals who give up during social hierarchy conflicts (Rantala et al., 2018). Still, feeling pain elicits an automatic fight or flight reaction, which temporarily blocks one’s sense of pain and, across our evolutionary history, has been useful for escaping from danger to safety (Wall, 1999). For this reason, although BDSM play may result in stress relief, studies have found that cortisol levels (i.e., physiological stress) rise as a result of BDSM play, particularly in submissives, while self-reported psychological stress decreases (Ambler et al., 2017; Sagarin et al., 2009). Similar results were found in a study observing participants in an extreme masochistic “Dance of Souls” ritual (Lee et al., 2016). In this ritual, dancers have weights hooked onto their skin, after which they dance for several hours in the beat of the drum. The ritual participants experienced a rise in physiological stress, but also a decrease in psychological stress and negative affect, and an increase in sexual arousal (Lee et al., 2016). Hence, the spike in cortisol is not necessarily an indication of distress. If there is an association of painful stimuli with sexual pleasure, the body’s parasympathetic system can counterbalance the stress response, resulting in relaxation, and this may play a pivotal role in the development of heightened sexual arousal in response to pain or the anticipation of pain.

One possible explanation for this is that individuals react differently to pain-induced physiological stress and interpersonally induced psychological stress. Physiological stress resulting from pain appears to reduce the amount of psychological stress experienced (Dunkley et al., 2020). During SM play, the submissive may also experience a state of altered consciousness similar to that which is experienced in Mindfulness practice, as pain may get a person to focus on the present moment and momentarily forget daily worries (Baumeister, 1988, 1997). Many of those who practice domination as their profession have reported that they function much like therapists and offer psychological care to their clients (Lindemann, 2011).

Whereas pain experienced in daily life, such as back pain or a broken limb, does not bring about pleasure and is just as discomforting and intensely experienced for SM-practitioners as others, pain administered during SM play can result in pleasure for these individuals (Dunkley et al., 2020). Why do individuals respond differently to pain administered during SM play than pain experienced in daily life? The explanation may lie in sexual arousal, which influences how pain is sensed. Experimental studies on pain administration have found that sexual arousal appears to increase one’s threshold for pain (Paterson et al., 2013; Whipple & Komisaruk, 1985). In one study, researchers found that vaginal stimulation to the point of orgasm doubled female’s pain threshold (Whipple & Komisaruk, 1985). Sexual interactions are known to activate the dopaminergic pathways of brain’s reward system, reducing one’s experience of negative sensations related to pain (Kender et al., 2008). It appears that sexual arousal combined with pain can result in individuals becoming conditioned to experience sexual pleasure from a certain type of pain. This type of sexual conditioning may explain why, for some, pain and strong stimuli during SM play can be enough to produce an orgasm (Easton & Hardy, 2001). This explains why SM play can be sexually pleasurable without necessarily including genitally oriented sexual interactions.

Another explanation for this difference lies in that one’s sense of pain can be influenced by whether the individual feels they have some control over it. In an experimental study in which pain was administered to research participants, their experience of pain was milder when they were able to control the cause of the pain (Weisenberg, 1977). SM pain is voluntary and usually within the control of the submissive, since they can stop the play any time via a safe-word. Contrastingly, back pain and pain from a broken limb is not in the control of the individual; thus, these types of pain are not experienced as pleasureful sensations.

The individual who administers the pain, such as whether the SM play is practiced with a complete stranger or someone the submissive knows, also influences the submissive’s sensitivity to pain. This was presented in a study in which married women were given electric shocks while the researchers measured their reactions via fMRI (Coan et al., 2006). The research participants who held their spouses’ hand self-reported much milder pain from the electric shocks compared to those who held the hand of a stranger and those who did not hold hands with anyone (Coan et al., 2006). The fMRI scans revealed that the brain region responsible for processing fear of pain was less activated when the participant’s spouse held her hand. Additionally, the better the couple rated their relationship, the stronger the spouse’s pain reducing effect was.

In a different study conducted with patients suffering from Fibromyalgia, which comes with long term and widespread pain, similarly found that patients’ pain experience was lessened in the presence of family or friends compared to when they were alone (Montoya et al., 2004). Therefore, it is appropriate to assume that if the dominant is an intimate partner, this influences the pain experienced during SM play. In one study utilizing survey data, submissives reported feeling more pleasure from pain when they felt that they were pleasing their partner (the dominant), which in turn added to the submissive’s experienced pleasure (Hebert & Weaver, 2015; Moser & Kleinplatz, 2007). When the dominating person is a trusted partner, and the submissive does not have to fear excessive pain, the submissive is better able to surrender and can more easily enter a state of altered consciousness (Dunkley et al., 2020). Hebert and Weaver (2015) found that SM play practice can indeed increase intimacy between the couple, improve communication skills, and increase feelings of trust.

Finally, Dahan (2019) has proposed that drawing a parallel between this altered consciousness and a similar pleasurable psychophysical altered state experienced by some during natural childbirth could explain how female sexual submission and masochism could offer adaptive benefits, particularly within the context of female reproductive processes. Considering the multifaceted nature of BDSM behaviors discussed above, we do not contend that BDSM practices in themselves are directly adaptive or conducive to greater reproductive success. However, in line with Dahan’s ideas, our exploration of sex differences in BDSM preferences suggests that these inclinations may stem from differences in individual choices and behaviors that potentially enhance reproductive success. While BDSM, as an extreme form of these preferences or inclinations, may not have inherent adaptive value, it could be regarded as an amplified expression of traits that hold adaptive value at more moderate levels.

## Discussion

Firstly, this paper discussed the possible roles of physiological factors, sexual conditioning, and sexual imprinting on BDSM interest and practice. In developmental psychology, the parent–child relationship and early childhood experiences are presumed key elements in individuals’ trajectories and adulthood outcomes, but we often hesitate to extend this thinking to sexual outcomes later in life. The evidence discussed above illustrates how these two biopsychosocial processes may contribute to some kinks across the heterogenous spectrum of BDSM. Yet, it seems that we can be more confident about the influence of these proximate processes on males’ sexual interests in BDSM than that of females. Secondly, this paper discussed the ultimate explanations surrounding BDSM power play and pain play, including role preferences and how pain can result in sexual pleasure. Biologically derived factors such as physiology and sexual orientation appear to be highly correlated with BDSM role preferences. The context in which strong stimuli are used and the resulting psychophysiological stress response also help to explain the pleasurable experiences yielded by BDSM practice.

The evolutionary explanations surrounding BDSM presented in this paper provide a contextual understanding of potential origins and ontogenesis of various kinks and interests. These are valuable for kink-identified individuals to recognize, as they may aid in BDSM practitioners’ understanding around, for example, kink interest discrepancy between partners (for more on this issue among BDSM-practicing consensually non-monogamous individuals, see Vilkin & Sprott, 2021). Additionally, these provide a particularly salient perspective for clinicians and professionals working with kink-identified individuals.

Over the course of the recent century, the BDSM and kink community have been pathologized, stigmatized, and attacked numerously. As unconventional as BDSM may be socially deemed, in examining the biopsychosocial and evolutionary explanations for BDSM interests and behaviors, we can identify these “natural” bases to BDSM. This does not give us the liberty to make moral or philosophical conclusions—as this would be based on a naturalistic fallacy and in violation of Hume’s law. However, it is fruitful to conceive how BDSM is not all that irregular in itself, and how aspects of BDSM exist and have existed across human history.

Thus, to perpetuate BDSM as pathological, when it is indeed a healthy form of intimacy for many (Wuyts & Morrens, 2022; Wuyts et al., 2020), can have harmful consequences in the therapeutic context. In such cases, it is possible that an erroneous association of kink or sexual fetishism with compulsion is partly to blame. Such sexual interests or behaviors—to the degree that one feels it disrupts their daily life and considers it a disorder—are unusual. Less than 1% of psychiatric patients report fetishism to be their main concern (Darcangelo, 2008). Only recently, the *International Classifications of Diseases and Related Health Problems* (ICD-11, published by the World Health Organization) removed sadomasochism from the list of paraphilic disorders on the basis that SM-practitioners usually participate in SM consensually (Moser, 2018). The overwhelmingly non-compulsive nature of BDSM interactions and the essentiality of consent within them are reason enough for kink-affirming clinical practice; understanding BDSM from an evolutionary perspective can help to demystify the subject and alleviate its stigmatization and pathologization (for further literature on de-pathologizing BDSM, see Ferenchak, 2022; Kolmes et al., 2006).

While stigma and misconceptions around the BDSM community persist, these evolutionary perspectives on BDSM interest and behavior offer a novel perspective through which the subject can be better understood. In this paper, we have presented a few of the human developmental processes and phenomena, resulting from natural selection over the course of our evolutionary history, that explain how BDSM behaviors and interests can arise in many individuals. This was far from a comprehensive review of the evolutionary perspectives on BDSM. Thus, we encourage social scientists to utilize evolutionary psychological frameworks to further investigate the topic in future literature and expand on this perspective of the subject.

## Data Availability

Not applicable.
